# Associations of improved air quality with lung function growth from childhood to adulthood: the BAMSE study

**DOI:** 10.1183/13993003.01783-2022

**Published:** 2023-05-04

**Authors:** Zhebin Yu, Simon Kebede Merid, Tom Bellander, Anna Bergström, Kristina Eneroth, Antonios Georgelis, Jenny Hallberg, Inger Kull, Petter Ljungman, Susanna Klevebro, Massimo Stafoggia, Gang Wang, Göran Pershagen, Olena Gruzieva, Erik Melén

**Affiliations:** 1 Institute of Environmental Medicine, Karolinska Institutet, Stockholm, Sweden; 2 Department of Clinical Sciences and Education, Södersjukhuset, Karolinska Institutet, Stockholm, Sweden; 3 Centre for Occupational and Environmental Medicine, Region Stockholm, Stockholm, Sweden; 4 SLB-analys, Environment and Health Administration, Stockholm, Sweden; 5 Sachs’ Children and Youth Hospital, Södersjukhuset, Stockholm, Sweden; 6 Department of Cardiology, Danderyd Hospital, Stockholm, Sweden; 7 Department of Epidemiology, Lazio Regional Health Service, Rome, Italy

## Abstract

**Background:**

The beneficial effect of improving air quality on lung function development remains understudied. We assessed associations of changes in ambient air pollution levels with lung function growth from childhood until young adulthood in a Swedish cohort study.

**Methods:**

In the prospective birth cohort BAMSE (Children, Allergy, Environment, Stockholm, Epidemiology (in Swedish)), spirometry was conducted at the 8-year (2002–2004), 16-year (2011–2013) and 24-year (2016–2019) follow-ups. Participants with spirometry data at 8 years and at least one other measurement in subsequent follow-ups were included (1509 participants with 3837 spirometry measurements). Ambient air pollution levels (particulate matter with diameter ≤2.5 μm (PM_2.5_), particulate matter with diameter ≤10 μm (PM_10_), black carbon (BC) and nitrogen oxides (NO*_x_*)) at residential addresses were estimated using dispersion modelling. Linear mixed effect models were used to estimate associations between air pollution exposure change and lung function development.

**Results:**

Overall, air pollution levels decreased progressively during the study period. For example, the median (interquartile range (IQR)) level of PM_2.5_ decreased from 8.24 (0.92) μg·m^−3^ during 2002–2004 to 5.21 (0.67) μg·m^−3^ during 2016–2019. At the individual level, for each IQR reduction of PM_2.5_ the lung function growth rate increased by 4.63 (95% CI 1.64–7.61) mL per year (p<0.001) for forced expiratory volume in 1 s and 9.38 (95% CI 4.76–14.00) mL per year (p<0.001) for forced vital capacity. Similar associations were also observed for reductions of BC and NO*_x_*. Associations persisted after adjustment for potential confounders and were not modified by asthma, allergic sensitisation, overweight, early-life air pollution exposure or dietary antioxidant intake.

**Conclusions:**

Long-term reduction of air pollution is associated with positive lung function development from childhood to young adulthood.

## Abstract 2

**Decrease in ambient air pollution exposure, even at relatively low levels, is associated with improvements in lung function growth from childhood to early adulthood** https://bit.ly/3jAFZTq

## Introduction

The adverse effects of ambient air pollution on children's respiratory health have been well documented [1, 2]. A large number of epidemiological studies support the relationship between long-term air pollution exposure and subsequent lung function impairment during childhood [1, 3–5]. In addition, a growing number of longitudinal studies utilising repeated lung function measurements suggest that air pollution exposure may also result in reduced lung function growth [6–9]. As portrayed in lung function growth trajectory studies [10, 11], lung function typically grows rapidly during the puberty period and reaches a peak in late teens for girls and early 20s for boys [12]. Either reduced level or reduced growth of lung function during childhood and adolescence could increase the risk of COPD in later adulthood [13].

Efforts have been made to reduce outdoor air pollution levels, but the beneficial effects of improved air quality on children's lung health are less understood [1, 14]. Most evidence of the beneficial effects of improved air quality comes from the extrapolation of dose–response relationships estimated in observational studies [15], while studies based on actual reduction in air pollution exposure remain scarce. In a subset (n=110) of the Children's Health Study (CHS), children who moved to areas with lower air pollution levels had larger lung function growth rate from age 10–11 to 15 years compared with those who moved to areas with higher air pollution levels [16]. More recent analyses in the CHS study showed that improved air quality in southern California in the USA was associated with improvements in lung function growth from 11 to 15 years of age among 2120 children [17]. However, the CHS studies evaluated community-level air pollution exposure and children were only followed up to around 15 years of age, which precluded analyses with peak lung function in adulthood. Thus, long-term studies are needed to provide empirical evidence and reduce the uncertainty of the beneficial effect of actual decline in individual-level air pollution exposure [18].

We have previously reported that traffic-related air pollution exposure in the first year of life was associated with reduced lung function levels measured by spirometry during childhood [19] and adolescence [6] in the BAMSE (Children, Allergy, Environment, Stockholm, Epidemiology (in Swedish)) birth cohort. In the current study, we extended the previous dataset with a new wave of spirometry measurements (2016–2019) and examined the association of improved air quality with lung function growth from childhood to young adulthood, as well as potential effect modification by sex, asthma, overweight, allergic sensitisation, dietary antioxidant intake, air pollution exposure in the first year of life and residential move.

## Methods

### Study population

The BAMSE study is an ongoing population-based cohort from Stockholm, Sweden, including 4089 newborns in 1994–1996. Details on study design, recruitment procedures and data collection have been provided elsewhere [20, 21]. Follow-ups were conducted at the ages of 1, 2, 4, 8, 12, 16 and 24 years. At the 8-, 16- and 24-year follow-ups, 2613 (64%), 2312 (57%) and 2039 (50%) participants, respectively, provided lung function data measured by spirometry. The present study population is comprised of participants with available spirometry data at 8 years and at least another spirometry at either 16 or 24 years (n=1509). This study was approved by the Swedish Ethical Review Authority and all participants, or their caregivers during childhood, gave written informed consent.

### Lung function measurements

Details of lung function measurements have been described elsewhere [22, 23]. In brief, spirometry was performed at the 8-year follow-up using a 2200 Pulmonary Function Laboratory spirometer (SensorMedics, Anaheim, CA, USA), at the 16-year follow-up using a Jaeger MasterScreen IOS system (CareFusion Technologies, San Diego, CA, USA) and at the 24-year follow-up using a Vyaire Vyntus system (Vyaire Medical, Mettawa, IL, USA). The same spirometry protocols were used at all follow-ups. All measurements were performed according to the American Thoracic Society (ATS)/European Respiratory Society (ERS) criteria [24]. We fitted linear regression models using sex, age and height to predict the forced expiratory volume in 1 s (FEV_1_) and forced vital capacity (FVC) values at each follow-up separately. Using this equation, the individual's residual values of FEV_1_ and FVC were calculated and used as the dependent variable in the main analysis. z-scores of FEV_1_ and FVC based on Global Lung Initiative (GLI) reference values were also calculated [25].

### Air pollution exposure assessment

Long-term exposures to particulate matter with diameter ≤2.5 μm (PM_2.5_), particulate matter with diameter ≤10 μm (PM_10_), black carbon (BC) and nitrogen oxides (NO*_x_*) were calculated at a grid of 35 m for addresses in densely populated areas and 100 or 500 m in less densely populated rural areas. In addition, a street canyon contribution was calculated for addresses located on busy inner-city streets flanked by contiguous high buildings using the Airviro street canyon model (until 2012; www.airviro.com/airviro/modules) and the OSPM operational street pollution model (from 2013 onwards; www.au.dk/OSPM). As input for the dispersion model, the historical emission inventories for 1990, 1995, 2000, 2011, 2015 and 2020 were used. For years in between, linear interpolation was used. The dispersion modelling is described in more detail in the supplementary material. Comparison of the calculated levels with measurements of annual mean values at a traffic monitoring site and two urban background sites (one for BC) resulted in R^2^-values of 0.99 for PM_2.5_, 0.99 for PM_10_, 0.98 for BC and 0.98 for NO*_x_* for the period 2002–2020 (2007–2020 for BC due to the availability of monitoring data). Annual average air pollution exposure preceding the date of spirometry measurement at the current address of follow-up was calculated. The difference in the annual average exposure between the 8-year follow-up and subsequent follow-ups was considered the main exposure index of improved air quality (supplementary figure E1). Time-weighted average air pollution exposure taking account of time spent at residential, daycare and school addresses (age 1–8, 8–16 and 16–24 years for each follow-up, respectively) was also used as an alternative exposure index.

In addition, short-term exposure was estimated based on measurement data from an urban background monitoring station located in central Stockholm to account for potential short-term effects of air pollution on lung function. Daily average concentrations on the day preceding lung function measurements were calculated for PM_2.5_, PM_10_ and NO*_x_*.

### Covariates

Information about covariates was obtained from the baseline questionnaire (sex, municipality at birth, parental education, parental occupation and maternal smoking during pregnancy), the 8-year questionnaire (antioxidant intake, asthma and environmental tobacco smoke), 16-year questionnaire (active smoking) and 24-year questionnaire (active smoking and education). Allergic sensitisation was based on IgE levels in blood at the 8-year follow-up [26]. Body mass index (BMI) was a time-varying variable calculated using the height and weight measured at each follow-up. Information on changing residential addresses after the 8-year follow-up was derived from the questionnaires and supplemented with the Swedish Tax Agency records. Full details of the definitions of the covariates are presented in the supplementary material.

### Statistical analysis

Descriptive analyses of the air pollution levels, spirometry variables and other covariates were performed initially. Correlations between improved levels of different air pollutants were estimated using the Spearman correlation index. We used a linear mixed effect model with both random intercept and random slopes for age, allowing for inter-individual differences of lung function at baseline and different rates of lung function growth during the follow-up period. Both residual values and GLI z-scores were modelled as the outcome. An interaction term of long-term improvement in air quality and age was added into the mixed effect model and the coefficient for this interaction term is the main estimate of interest, which can be interpreted as the association of a 1-unit increment of improved air quality with annual rate of growth in lung function.

Covariates adjusted in the mixed effect model were determined *a priori* and included: parental occupation at birth, parental education level at birth, municipality at birth, maternal smoking during pregnancy, air pollution exposure during the first year of life, environmental tobacco smoke at 8 years, and time-varying covariates including BMI at 8, 16 and 24 years, active smoking at 16 and 24 years, and education level at 24 years. Change of air pollution exposure was modelled as a continuous variable assuming a linear exposure–response relationship and estimates with corresponding 95% confidence intervals were presented as per interquartile range (IQR) change.

Subgroup analyses were conducted to test whether the effect size differed by sex, asthma status, overweight, allergic sensitisation at 8 years, air pollution exposure during the first year of life (high or low based on median values), antioxidant intake (high or low based on median values) and moving after the 8-year follow-up, and interaction was tested by adding exposure-modifier interaction terms into the model. We also investigated the association of air pollution with lung function growth in different periods (*i.e.* from 8 to 16 years and 16 to 24 years modelled separately). In addition, we explored the association between improved air quality and lower limit of normal (LLN) z-scores of FEV_1_ and FVC (defined as < −1.645sd) using a generalised linear mixed effect model.

As for sensitivity analyses, we: 1) ran the analysis based on the relative change of air pollution as exposure; 2) additionally adjusted for daily air pollution concentrations 1 day prior to the date of spirometry measurement; 3) used time-weighted average air pollution exposure since the date of the previous follow-up with the lung function measurement to calculate the improved air quality and modelled as the exposure; 4) replaced the 24-year follow-up data with the most recent coronavirus disease 2019 (COVID-19) follow-up data from 2020–2021 (mean age 25.7 years) (described in detail in the supplementary material) [27, 28].

All analyses were performed using R version 4.0.5 (www.r-project.org), with two-sided p-values <0.05 indicating statistical significance.

## Results

The basic characteristics of the included participants are presented in table 1. The mean±sd age at each follow-up was 8.3±0.5, 16.7±0.4 and 22.6±0.6 years, respectively. Anthropometry data, lung function raw values and z-scores at each follow-up are presented in table 2. Comparisons between the included study sample and the full cohort are presented in supplementary table E1.

**TABLE 1:** Characteristics of the study population at the 8-, 16- and 24-year follow-ups in the BAMSE cohort for subjects with at least one follow-up after age 8 years

	**8-year follow-up**	**16-year follow-up**	**24-year follow-up**
**Participants, n**	1509	1224	1104
**Age, years**	8.3±0.5	16.7±0.4	22.6±0.6
**Sex**			
Male	721 (47.8)	554 (45.3)	486 (44.0)
Female	788 (52.2)	670 (54.7)	618 (56.0)
**Parental occupation at birth**			
White collar worker	1301 (86.2)	1064 (86.9)	957 (86.7)
Blue collar worker	208 (13.8)	160 (13.1)	147 (13.3)
**Parental education at birth**			
Elementary school	28 (1.8)	18 (1.5)	22 (2.0)
High school	635 (42.1)	512 (41.8)	448 (40.6)
University	846 (56.1)	694 (56.7)	634 (57.4)
**Education at 24** **years**			
Elementary school or high school			405 (36.7)
University or college			699 (63.3)
**Maternal smoking during pregnancy**	179 (11.9)	136 (11.1)	125 (11.3)
**Environmental tobacco smoke**	256 (17.0)	141 (11.5)	27 (2.4)
**Active smoking**		147 (12.0)	226 (20.5)
**Asthma at age 0–8** **years**	237 (15.7)	198 (16.2)	159 (14.4)
**Allergic sensitisation at 8** **years**	519 (34.4)	431 (35.2)	381 (34.5)
**Moved after 8** **years**	1194 (79.1)	955 (78.0)	811 (73.5)
**Short-term air pollution exposure^#^**			
PM_2.5_, μg·m^−3^	8.6±5.9	4.5±4.3	4.6±2.6
PM_10_, μg·m^−3^	17.4±10.6	15.5±8.5	12.2±7.4
NO*_x_*, μg·m^−3^	23.0±12.9	14.9±10.4	15.4±9.1

Data are presented as n, mean±sd or n (%). ^#^: daily air pollutant concentrations at the urban background monitoring station 1 day prior to the spirometry measurement.

**TABLE 2:** Distribution of height, weight, body mass index (BMI), forced expiratory volume in 1 s (FEV_1_) and forced vital capacity (FVC) for the participants

	**8-year follow-up (n=1509)**		**16-year follow-up (n=1224)**		**24-year follow-up (n=1104)**	
	**Male (n=721)**	**Female (n=788)**	**Male (n=554)**	**Female (n=670)**	**Male (n=486)**	**Female (n=618)**
**Height, m**	1.33±0.06	1.32±0.06	1.79±0.07	1.67±0.06	1.82±0.07	1.69±0.06
**Weight, kg**	30.28±5.14	29.96±5.24	70.49±11.14	60.95±8.69	78.39±13.39	64.64±10.95
**BMI, kg·m^−2^**	17.12±2.01	17.17±2.14	21.88±3.16	21.73±2.85	23.59±3.71	22.75±3.77
**Lung function**						
FEV_1_, mL	1822.39±278.98	1726.83±250.95	4485.34±654.47	3482.79±423.46	4720.98±643.79	3507.31±415.63
FVC, mL	2147.95±339.19	1983.49±291.22	5397.22±782.79	4052.12±499.20	5833.65±829.37	4161.54±513.31
FEV_1_ z-score^#^	0.37±0.92	0.45±0.93	−0.05±0.97	−0.05±0.88	−0.37±0.92	−0.17±0.83
FVC z-score^#^	0.58±0.91	0.61±0.90	0.18±0.96	0.18±0.86	−0.10±0.88	0.03±0.84

Data are presented as mean±sd. ^#^: z-score was calculated based on the reference equation from the Global Lung Initiative 2012 [25].

Overall, long-term air pollution exposure levels decreased over the study period (table 3), but not all participants experienced a reduction of air pollution exposure (supplementary table E2). The median level of PM_2.5_ declined from 8.24 μg·m^−3^ at the 8-year follow-up to 5.21 μg·m^−3^ at the 24-year follow-up, with the individual range of reduction from −0.50 to 6.12 μg·m^−3^ (positive value indicating reduction). A similar overall decline trend was observed for other air pollutants, with the range of reduction from −7.64 to 10.9 μg·m^−3^ for PM_10_, −0.19 to 1.78 μg·m^−3^ for BC and −44.4 to 48.40 μg·m^−3^ for NO*_x_*. The distribution of air pollution exposure was similar for the included participants compared with those with available air pollution data in the full cohort (supplementary table E3). A similar decline trend of air pollution levels was observed using time-weighted average exposure since the preceding follow-up with the spirometry measurement (supplementary table E4). Concurrent estimates for change of PM_2.5_, PM_10_, BC and NO*_x_* were highly correlated (Spearman correlation index 0.76–0.98 for 8 to 16 years and 0.73–0.92 for 8 to 24 years) (supplementary table E5).

**TABLE 3:** Annual average air pollution exposure preceding each follow-up during the study period

	**8-year follow-up**	**16-year follow-up**	**24-year follow-up**
**PM_2.5_, μg·m^−3^**	8.24 (0.92)	6.63 (0.62)	5.21 (0.67)
**PM_10_, μg·m^−3^**	13.60 (2.48)	13.30 (1.82)	11.60 (1.47)
**BC, μg·m^−3^**	0.79 (0.45)	0.63 (0.25)	0.38 (0.10)
**NO*_x_*, μg·m^−3^**	17.00 (14.10)	10.40 (9.40)	9.93 (7.65)
**Reduction from 8-year follow-up^#^**			
PM_2.5_			
Absolute, μg·m^−3^		1.65 (0.57)	3.01 (0.85)
Relative, %		20.6 (5.7)	36.5 (8.3)
PM_10_			
Absolute, μg·m^−3^		0.30 (1.34)	1.84 (1.70)
Relative, %		2.3 (9.2)	13.6 (10.7)
BC			
Absolute, μg·m^−3^		0.14 (0.26)	0.39 (0.38)
Relative, %		18.6 (21.4)	51.9 (19.7)
NO*_x_*			
Absolute, μg·m^−3^		5.44 (6.64)	5.98 (8.51)
Relative, %		34.5 (13.9)	40.3 (30.4)

Data are presented as median (interquartile range). PM_2.5_: particulate matter with diameter ≤2.5 μm; PM_10_: particulate matter with diameter ≤10 μm; BC: black carbon; NO*_x_*: nitrogen oxides. ^#^: absolute air pollution change (*i.e.* reduction) between the 8- and 16-year follow-ups calculated as concentration at 8 years minus concentration at 16 years, and between the 8- and 24-year follow-ups calculated as concentration at 8 years minus concentration at 24 years. Relative change calculated as absolute change/concentration at 8 years×100%. Positive values for reduction from the 8-year follow-up indicate improved air quality in the later period.

Reduced levels of air pollution were associated with increased growth rate in both FEV_1_ and FVC (table 4). We found the mean growth rate in FEV_1_ increased by 4.63 (95% CI 1.64–7.61) mL per year per decrease of 2.19 μg·m^−3^ in PM_2.5_, by 0.72 (95% CI −0.91–2.35) mL per year per decrease of 1.00 μg·m^−3^ in PM_10_, by 2.80 (95% CI 0.66–4.93) mL per year per decrease of 0.28 μg·m^−3^ in BC and by 1.70 (95% CI −0.16–3.57) mL per year per decrease of 6.17 μg·m^−3^ in NO*_x_*. Positive associations were also observed when modelling the GLI z-scores of FEV_1_ as outcome. Similar associations and larger effect sizes with decreased air pollution levels were observed for FVC.

**TABLE 4:** Association between improvement of air quality and differences in lung function growth from age 8 to 24 years

	**Unit of improvement in exposure**	**Raw value**		**GLI z-score**	
		**Difference in FEV_1_ growth, mL per year (95% CI)^#^**	**Difference in FVC growth, mL per year (95% CI)^#^**	**Difference in FEV_1_ growth, sd per year (95% CI)**	**Difference in FVC growth, sd per year (95% CI)**
**PM_2.5_**	2.19 μg·m^−3^	4.63 (1.64–7.61)	9.38 (4.76–14.00)	0.03 (0.02–0.04)	0.04 (0.03–0.05)
**PM_10_**	1.00 μg·m^−3^	0.72 (−0.91–2.35)	2.77 (0.19–5.35)	0.01 (0.00–0.02)	0.01 (0.01–0.02)
**BC**	0.28 μg·m^−3^	2.80 (0.66–4.93)	5.59 (2.30–8.87)	0.02 (0.01–0.03)	0.02 (0.01–0.03)
**NO*_x_***	6.17 μg·m^−3^	1.70 (−0.16–3.57)	3.29 (0.35–6.23)	0.01 (0.01–0.02)	0.01 (0.01–0.02)

GLI: Global Lung Initiative; FEV_1_: forced expiratory volume in 1 s; FVC: forced vital capacity; PM_2.5_: particulate matter with diameter ≤2.5 μm; PM_10_: particulate matter with diameter ≤10 μm; BC: black carbon; NO*_x_*: nitrogen oxides. **^#^**: estimates were adjusted for age, sex, height, body mass index (BMI) at age 8 years, municipality at birth, parental education level at birth, parental occupation at birth, maternal smoking during pregnancy, environmental tobacco smoke at 8 years, air pollution exposure during the first year of life, BMI at 16 and 24 years, active smoking at 16 and 24 years, and education level at 24 years. Estimates were interpreted as the difference in 1-year growth (95% CI) in FEV_1_ and FVC for per unit improvement of air pollution concentrations, with positive values indicating positive associations between improved air quality and increased rate of FEV_1_ or FVC growth.

The beneficial effects of reduced air pollution on FEV_1_ and FVC growth were robust in sensitivity analysis including using the relative change of air pollution as the exposure, further adjustment for short-term air pollution exposure, as well as using average exposure between the follow-ups and using the most recent COVID-19 follow-up data collected in 2020–2021 (supplementary table E6) [27].

**FIGURE 1:**
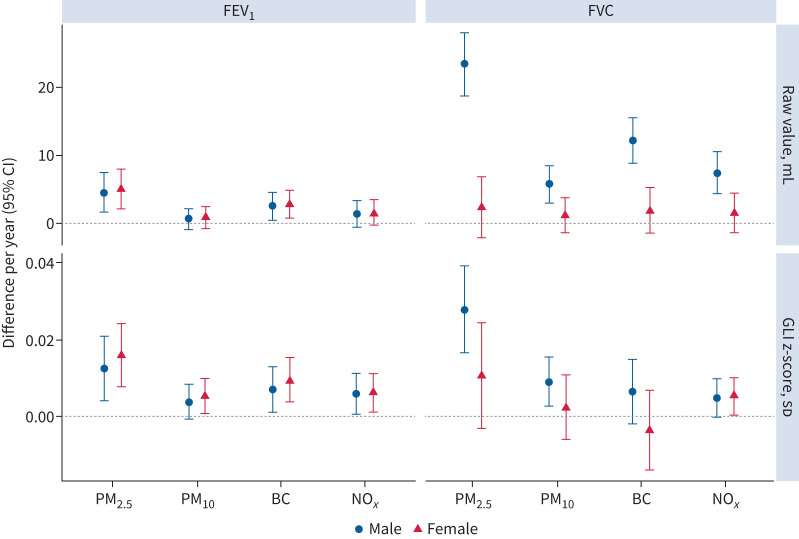
Sex-specific associations between improvement of air quality and differences in forced expiratory volume in 1 s (FEV_1_) and forced vital capacity (FVC) growth from age 8 to 24 years. Results were adjusted for age, height, body mass index (BMI) at age 8 years, municipality at birth, parental education level at birth, parental occupation at birth, maternal smoking during pregnancy, environmental tobacco smoke at 8 years, air pollution exposure during the first year of life, BMI at 16 and 24 years, active smoking at 16 and 24 years, and education level at 24 years. Estimates were interpreted as the difference in 1-year growth (95% CI) in FEV_1_ and FVC for per unit improvement of air pollution concentrations, with positive values indicating positive associations between improved air quality and increased rate of FEV_1_ or FVC growth. GLI: Global Lung Initiative; PM_2.5_: particulate matter with diameter ≤2.5 μm; PM_10_: particulate matter with diameter ≤10 μm; BC: black carbon; NO*_x_*: nitrogen oxides.

In subgroup analyses, significantly larger effects were observed among males in FVC growth rate compared with females but not FEV_1_ growth rate (figure 1). No effect modification was observed for asthma, overweight, sensitisation, antioxidant intake and early-life air pollution exposure on both FEV_1_ growth (supplementary figure E2) and FVC growth (supplementary figure E3). The change in air pollution levels for movers and non-movers was largely similar (supplementary table E7), and no estimate differences were observed (supplementary figures E2 and E3). When we modelled the two consecutive follow-ups separately, substantially larger effects of reduced air pollution on lung function growth were observed from childhood (8 years) to adolescence (16 years), although positive associations were also observed from adolescence (16 years) to young adulthood (24 years) (supplementary table E8). Protective associations were observed for reduction of PM_2.5_ and having lung function below the LLN at 16 and 24 years (OR 0.90 (95% CI 0.84–0.97) for FEV_1_ and OR 0.78 (95% CI 0.70–0.86) for FVC) (supplementary table E9).

## Discussion

In this population-based prospective study, we found that more rapidly declining concentrations of PM_2.5_, BC and NO*_x_* were consistently associated with increased annual growth rate of both FEV_1_ and FVC. These associations were not modified by asthma, overweight, allergic sensitisation, dietary antioxidant intake or early-life exposures, suggesting a general beneficial effect from improvements in air quality.

We previously reported inverse associations between traffic-related air pollution (PM_10_ and NO*_x_*) during the first year of life and FEV_1_ values at 8 and 16 years of age [6, 19, 29]. The current study extends the previous findings that improved air quality in later ages, especially during the pubertal period, has a beneficial effect on lung function development, regardless of the level of air pollution exposure during the first year of life. Consistent with our analysis, there are panel studies showing that relocating to areas with lower air pollution levels was associated with increased lung function levels [30]. To the best of our knowledge, so far only the CHS study [17] reported the positive association between reduced air pollution over two decades and improved lung function development from 11 to 15 years of age. Despite the differences in study design, exposure assessment and age range of the study population, our findings concur with the results from the CHS study, with the exception that the associations of reduction in PM_10_ were less clear than those of PM_2.5_ and BC A potential explanation might be that the reduction of PM_10_ concentrations in our study was small during the study period, which limited the power of our analysis. The reduction of absolute concentrations of BC was also small, yet significant associations with lung function growth were observed. Sensitivity analysis using the relative change of air pollution exposure from the 8-year follow-up showed similar associations (supplementary table E6), suggesting it is important to take into account the background concentrations when comparing the beneficial effects of reduction in exposure between different air pollutants.

One extension of the current study is by including spirometry measurements at the 24-year follow-up (and one more follow-up at mean age 25.7 years during the COVID-19 pandemic as part of the sensitivity analyses). Our study added evidence to the knowledge gap that the beneficial effect of improved air quality on lung function still existed from adolescence to young adulthood, although the effect size in this period is much smaller than that of the puberty period. Additional longitudinal studies are warranted to confirm these findings that appear very important for preventive and management strategies. Reduced lung function in adulthood is known to be associated with increased risk of subsequent respiratory morbidity [31], premature death [32, 33] and other health outcomes [34, 35] in later life, suggesting potentially greater benefits in reducing disease burden from improved air quality.

We observed significant associations between reduced air pollution and increased lung function growth in Sweden where air pollution levels are generally lower compared with many cities worldwide (*e.g.* annual mean concentrations of PM_2.5_ in 2019 were 5.9 μg·m^−3^ in Stockholm, 9.0 μg·m^−3^ in Los Angeles, 15.5 μg·m^−3^ in Paris, 37.0 μg·m^−3^ in Beijing and 105 μg·m^−3^ in Delhi [36]), and observed associations remained significant across different sensitivity analyses. Consistent with our analysis, Chen *et al.* [37] found that a decrease in long-term PM_2.5_ exposure due to relocating was associated with lower mortality at relatively low levels of PM_2.5_ in Canada. Analyses from the Health Effect Institute (Boston, MA, USA), with a particular focus on low-exposure settings [38–40], underscore the need for continuously reducing ambient air pollution. Road traffic is the primary source of air pollution in the current study region [41]. Owing to the high correlation between the change of different air pollutants in the same period, we are unable to disentangle the independent effect of each air pollutant. However, our results indicate that broad-band efforts targeting traffic-related pollution to improve general air quality may achieve public health benefits.

We observed larger growth rate of FVC among males compared with females, which is consistent with the CHS study [17] and our previous BAMSE study [6], although the evidence regarding a possible air pollution×sex interaction is rather mixed [3]. Different natural growth rate, pubertal staging and sex-switch of asthma may need to be considered in future studies investigating the air pollution×sex interaction on lung function. In the current study, we observed a beneficial effect on lung function growth from improved air quality among both overweight children and normal weight children. A previous study from the SAPALDIA (Swiss Cohort Study on Air Pollution and Lung Diseases in Adults) cohort reported that improved air quality was associated with attenuated decline rates in lung function among low and normal BMI adults, but not overweight or obese adults [42]. However, a study from the SALIA (Study on the influence of Air pollution on Lung function, Inflammation and Aging) cohort found no effect modification by BMI between improved air quality and change in lung function [43]. The different age ranges in these studies preclude comparability between the studies. Evidence regarding the effect modification by asthma, sensitisation and antioxidant intake is also scarce [1, 3]. Identifying potential modifiers for the association between air pollution and lung function growth remains to be addressed by future studies, ideally from collaboration of multi-cohorts to provide enough statistical power.

### Strengths and limitations

The strengths of our study were the long-term follow-up with the availability of three repeated spirometry measurements from childhood until young adulthood, use of high-resolution spatiotemporal air pollution modelling to calculate the change of air pollution exposure on an individual level, and detailed information about potential confounders and effect modifiers. One potential limitation is that different spirometers were used at 8, 16 and 24 years, but all devices met the ERS/ATS recommendations. Since the same spirometers and examination protocols were used for all individuals in the respective follow-up, we believe that any bias due to the use of different of spirometers would be non-differential on the association estimates. Some exposure misclassifications may also exist as we lack detailed individual information of time activity and indoor exposure. Selection bias may be of concern, but the comparison between the included participants and the full cohort showed only minor differences in distribution of background characteristics and very similar air pollution exposure over the study period. In addition, other time-varying environmental exposures may act as potential confounders, such as noise and surrounding greenness, although less is known about the relationship between these exposures and lung function growth. We acknowledge that we cannot exclude the possible existence of unmeasured time-varying confounding factors that may co-vary with air pollution reduction and have associations with observed lung function change. We were also only able to assess the association between absolute change of air pollution levels and lung function growth, while variability of the change of particulate matter chemical composition is not captured in the current analysis.

### Conclusions

Our results indicate that a decrease in ambient long-term air pollution levels, even at the relatively low levels in Stockholm, Sweden, was associated with improvements in lung function growth, supporting efforts to continuously improve air quality.

## Supplementary material

10.1183/13993003.01783-2022.Supp1 **Please note:** supplementary material is not edited by the Editorial Office, and is uploaded as it has been supplied by the author.
Supplementary material ERJ-01783-2022.Supplement

## Shareable PDF

10.1183/13993003.01783-2022.Shareable1 This one-page PDF can be shared freely online.
Shareable PDF ERJ-01783-2022.Shareable
