# Supplementary material for: Associations of improved air quality with lung function growth from childhood to adulthood: the BAMSE study
Source: Eur Respir J. 2023 May 4;61(5):2201783. doi: 10.1183/13993003.01783-2022 (PMC10160798; doi:10.1183/13993003.01783-2022)
Supplement: Supplementary file 1 [file ERJ-01783-2022.Supplement.pdf]

Associations of improved air quality with lung function growth from childhood to adulthood: The BAMSE study

Zhebin Yu, Simon Kebede Merid, Tom Bellander, Anna Bergström, Kristina Eneroth, Antonios Georgelis, Jenny Hallberg, Inger Kull, Petter Ljungman, Susanna Klevebro, Massimo Stafoggia, Gang Wang, Göran Pershagen, Olena Gruzieva, Erik Melén

Online supplemental materials

eMethods

Supplementary Table E1 Distributions of selected characteristics among all participants in the cohort and the participants included in the present study

Supplementary Table E2 Distribution of reduction in air pollution exposure during the study period

Supplementary Table E3 Comparison of air pollution exposure levels between included participants and all participants with available air pollution exposure data

Supplementary Table E4 Distribution of 8-year cumulative air pollution exposure during the study period

Supplementary Table E5 Spearman correlations between reduction of air pollutants between age 8 to age 16 and age 8 to age 24

Supplementary Table E6 Sensitivity analyses of the association between air quality improvement and differences in lung function growth from age eight to age 24 years

Supplementary Table E7 Distribution of the reduced air pollution exposure during the study period stratified based on moving after 8-year follow-up

Supplementary Table E8 Association between improvement of air quality and differences in lung function growth stratified by study period

Supplementary Table E9 Odds ratios for the associations between improved air quality and lower limit of normal (LLN) z-scores of FEV1 and FVC at 16-years and 24-years of age

Supplementary Figure E1 Flowchart of the study timeline

Supplementary Figure E2 Subgroup analyses of the association between improvement of air quality and differences in FEV_1 growth from age eight to age 24.

Supplementary Figure E3 Subgroup analyses of the association between improvement of air quality and differences in FVC growth from age eight to age 24

eMethods

Study population

Starting August 1, 2020, participants who attended both questionnaire and clinical examination at 24-year follow-up (N=2270) were invited to a new COVID-19 follow-up[1, 2], which included a web questionnaire from August to November 2020 (phase 1), a clinical examination and a web questionnaire from October 2020 to June 2021 (phase 2), and a web questionnaire from August 2021 to February 2022 (phase 3). During the phase 2 clinical examination, lung function assessment and measurements of antibody against SARS-CoV-2 were conducted. Lung function was measured (according to European Respiratory Society/American Thoracic Society guidelines[3]) by using a Vyaire Vyntus spirometer (Vyaire Medical, Chicago, Ill) to determine FEV1 and FVC values. A total of 593 participants (mean (range) age: 25.7 (23.9-27.2)) had lung function measurements at 8-year follow-up and were included in the sensitivity analysis in Table E6. Infection with SARS-CoV-2 was not adjusted in this sensitivity analysis as our recent analysis found no evidence of mild-to-moderate COVID-19 affecting lung function in the same cohort[4].

Air pollution Exposure Assessment

The annual mean concentrations of $PM_{2.5}$, PM_{10}, black carbon (BC) and nitrogen oxides (NO_x) at the individual-level addresses were calculated using a Gaussian air quality dispersion model and a wind model, both part of the Airviro air quality management system (https://www.airviro.com/airviro/). The Gaussian model calculates the horizontal distribution of air pollution concentrations 2 m above ground level. In densely populated areas, the calculations represent concentrations 2 m above roof level. The calculations use a variable grid size, between 35 × 35 m and 500 × 500 m, with the smallest grid size in the vicinity of roads and chimneys. In addition, a street canyon contribution was calculated for addresses located on busy inner-city streets flanked by contiguous high buildings using the Airviro street canyon model (until the year 2012; https://www.airviro.com/airviro/modules/) and the Operational Street Pollution Model, OSPM (from 2013 onwards; www.au.dk/OSPM). Meteorological data for the wind model were taken from a 50-m mast in Högdalen in southern Stockholm. As input to the dispersion modelling, emission inventories for the years 1990, 1995, 2000, 2011, 2015 and 2020 were used. For years in between linear regression was used. The emission inventories include local emissions from road traffic (exhaust and non-exhaust), residential wood combustion, energy production, industrial processes, and other sources (eg, off-road machinery and agriculture) in Stockholm and Uppsala counties, and are described in detail

elsewhere[5]. In the Stockholm region, road traffic is the dominant local source of $PM_{2.5}$, PM_{10}, BC and NO_x. Road traffic emissions of NO_x and PM-exhaust for different vehicle types, speeds and driving conditions were calculated according to the European emission model HBEFA (https://www.hbefa.net/e/index.html). Emission factors for BC were estimated based on the fraction of BC in PM-exhaust. This fraction varies depending on fuel and type of vehicle and the information was derived from the Transphorm project (http://www.transphorm.eu/). Emission factors for non-exhaust PM, including road wear and some contributions from brake and tire wear, were obtained from Omstedt et al. (2005)[6] and from the NORTRIP model[7, 8]. In Stockholm during the late winter and in connection with dry road surfaces, the contribution from studded tire wear can be 80 % to 90 % of the total PM_{10} levels. The road wear PM also makes a considerable contribution to the total $PM_{2.5}$ levels. To obtain total air pollution concentrations, annual average long-range contributions based on continuous measurements at regional background stations, were added to the locally modelled concentrations. Due to low availability of monitored BC (the measurements started in 2007), and observed low year-to-year variability in existing measurement data of the related metric black smoke, the long-range transport contributions of BC were assumed constant over the period 1990 – 2012, i.e. 0.3 $\mu g/m^3$. For NO_x, measurement data were available from 1994. The long-distance contribution for previous years has been estimated on the basis of extrapolation of measurement data back in time. For PM_{10} and $PM_{2.5}$, long-distance contributions from 2006 onwards consist of measured concentrations at a background monitoring station approx. 50 km northeast of Stockholm. For the years before 2006, the long-distance contributions of PM_{10} and $PM_{2.5}$ have been estimated by combining measurement data from two regional background stations and its relationship to measured concentrations at an urban background station in central Stockholm. Age- and municipality-specific information on the time children were at day care and school facilities was used to estimate the time-weighted average exposure.

Definitions of covariates

Covariates	Definition
Parental occupation at birth	Socioeconomic status for the household according to dominance

	order in three classes. (0= Blue collar worker, 1= white collar worker (including Liberal professional practician with university graduate jobs)
Parental education at birth	Based on maternal and paternal education level in three categories (1= elementary school, 2=high school, 3= University).
Education at 24 years	Based on the education level at the time of 24-year follow-up (1= elementary or high school;2= university or college)
Maternal smoking during pregnancy	The mother smoked at least one cigarette per day at the time of questionnaire 0
Environmental tobacco smoke	Any of the parents smoked daily at the time of questionnaire 8 or 16 or indoor exposure to tobacco smoke (at home, at work and/or indoor at another place) daily
Active smoking	Current smoking at the time of questionnaire 16 or 24 years
Asthma at age 1-8 years	Fulfilling the asthma definition developed within the Mechanisms of the Development of Allergy[9] consortium over time up to 8 years. Asthma is defined based on at least 2 of the 3 following criteria: (1) symptoms of wheezing in the last 12 months before the date of questionnaire follow-up; (2) ever having a physician's diagnosis of asthma; (3) asthma medicine used occasionally or regularly in the last 12 months.
Allergic sensitization at 8 years	The blood samples taken at 8-years of age were analyzed with Phadiatop (a mixture of common airborn allergens: birch, timonthy, mugwort, cat, dog, horse, mold and house dust mite) and fx5 (a mixture of common food allergens: cow's milk, egg white, soy bean, peanut, cod fish and wheat)[10](ImmunoCAP System, Phadia AB, Uppsala, Weden). IgE value equal to or greater than 0.35 kU/L were regarded as positive.
Total antioxidant capacity (TAC) of the diet at 8 years	Individual TAC estimates were obtained by combining the information on frequency of consumption of specific food items with information from a database of common foods analysed with the oxygen radical

	absorbance capacity (ORAC) method on the average ORAC content (µmol Trolox equivalents (TE) per day) of age-specific portion sizes[11]. We subsequently divided the TAC into low and high using the median value as cut-off.
Moving	Any residential address change after the date of 8-year follow-up

Supplementary Table E1 Distributions of selected characteristics among all participants in the cohort and the participants included in the present study

	Full cohort (N=4089)	Included participants (N=1509)
Male	2065/4089 (50.5)	721/1509 (47.8)
White collar work of parents at birth	3323/4089 (82.7)	1301/1509 (86.2)
Highest education of the household at birth		
Elementary school	107/4082 (2.6)	28/1509 (1.9)
High school	1814/4082 (44.4)	635/1509 (42.1)
University	2161/4082 (52.9)	846/1509 (56.1)
Maternal smoking during pregnancy	527/4088 (12.9)	179/1509 (11.9)
Environmental tobacco smoke at 8 yrs	597/3382 (17.7)	256/1509 (17.0)
Asthma at 0-8 yrs	470/3238 (14.5)	237/1509 (15.7)
Allergic sensitization at 8 yrs	851/2447 (34.8)	519/1509 (34.4)
Active smoking at 16 yrs	373/3108 (12.0)	147/1509 (12.0)
Active smoking at 24 yrs	633/3056 (26.1)	226/1509 (20.5)

Supplementary Table E2 Distribution of reduction in air pollution exposure during the study period

	Min	5th	25th	50th	75th	95th	Max
	16-year follow-up						
Reduction of $PM_{2.5}$ from 8-year*, μg/m^3	-1.88	0.68	1.41	1.66	1.97	2.69	4.36
Reduction of PM_{10} from 8-year*, μg/m^3	-6.66	-1.26	-0.25	0.32	1.08	2.84	9.45
Reduction of BC from 8-year*, μg/m^3	-0.78	-0.04	0.05	0.14	0.32	0.66	1.51
Reduction of NO_x from 8-year*, μg/m^3	-26.80	0.35	3.40	5.53	10.00	19.77	49.60
	24-year follow-up						
Reduction of $PM_{2.5}$ from 8-year*, μg/m^3	-0.50	1.87	2.56	3.00	3.43	4.29	6.12
Reduction of PM_{10} from 8-year*, μg/m^3	-7.64	-1.08	1.01	1.80	2.71	4.85	10.9
Reduction of BC from 8-year*, μg/m^3	-0.19	0.09	0.25	0.38	0.64	1.03	1.78
Reduction of NO_x from 8-year*, μg/m^3	-44.4	-8.83	2.75	5.72	11.30	22.29	48.40

$PM_{2.5}$, particulate matter with diameter ≤ 2.5 μm; PM_{10}, particulate matter with diameter ≤ 10 μm; BC, black carbon; NOx, nitrogen oxides.

*: air pollution change between the 8-year and 16-year follow-up (concentration at 8-year – concentration at 16-year) and between 8-year and 24-year follow-up (concentration at 8-year – concentration at 24-year), with positive values indicating improvement of air quality.

Supplementary Table E3 Comparison of air pollution exposure levels between included participants and all participants with available air pollution exposure data

	Included participants			All participants with available air pollution data		
	8-year follow-up (n=1509)	16-year follow-up (n=1224)	24-year follow-up (n=1104)	8-year follow-up (n=3060)	16-year follow-up (n=2812)	24-year follow-up (n=1856)
$PM_{2.5}$	8.2 (0.92)	6.63 (0.62)	5.22 (0.67)	8.2 (0.93)	6.63 (0.63)	5.21 (0.66)
PM_{10}	13.6 (2.48)	13.3 (1.82)	11.6 (1.47)	13.5 (2.51)	13.2 (1.86)	11.6 (1.47)
Black carbon	0.78 (0.45)	0.64 (0.25)	0.37 (0.10)	0.79 (0.46)	0.63 (0.23)	0.374 (0.10)
NO_x	17.0 (14.1)	10.4 (9.4)	9.90 (7.65)	16.8 (14.2)	10.3 (9.11)	9.71 (7.36)
Reduction of $PM_{2.5}$*	-	1.65 (0.57)	3.01 (0.85)		1.63 (0.57)	2.99 (0.86)
Reduction of PM_{10}*	-	0.30 (1.34)	1.84 (1.70)		0.35 (1.45)	1.83 (1.71)
Reduction of BC*	-	0.14 (0.26)	0.39 (0.38)		0.135 (0.26)	0.396 (0.39)
Reduction of NO_x*	-	5.44 (6.64)	5.98 (8.51)		5.34 (6.5)	5.98 (8.55)

Data were presented as median (interquartile range). $PM_{2.5}$, particulate matter with diameter ≤ 2.5 μm; PM_{10}, particulate matter with diameter ≤ 10 μm; BC, black carbon; NOx, nitrogen oxides.

*: air pollution changes between the 8-year and 16-year follow-up (concentration at 8-year – concentration at 16-year) and between 8-year and 24-year follow-up (concentration at 8-year – concentration at 24-year), with positive values indicating improvement of air quality.

Supplementary Table E4 Distribution of 8-year cumulative air pollution exposure during the study period

Air pollution concentrations	8-year follow-up	16-year follow-up	24-year follow-up
$PM_{2.5}$	7.95 (0.95)	7.46 (0.74)	5.46 (0.54)
PM_{10}	13.40 (2.55)	12.90 (2.13)	12.70 (1.69)
BC	0.86 (0.48)	0.67 (0.32)	0.46 (0.149)
NO_x	21.50 (16.32)	12.30 (11.12)	11.60 (9.34)
Reduction of $PM_{2.5}$ from 8-year*	-	0.47 (0.52)	2.49 (0.68)
Reduction of PM_{10} from 8-year*	-	0.39 (1.08)	0.53 (1.33)
Reduction of BC from 8-year*	-	0.14 (0.241)	0.38 (0.36)
Reduction of NO_x from 8-year*	-	6.60 (7.89)	7.63 (8.33)

$PM_{2.5}$, particulate matter with diameter ≤ 2.5 µm; PM_{10}, particulate matter with diameter ≤ 10 µm; BC, black carbon; NOx, nitrogen oxides.

*: air pollution changes between the 8-year and 16-year follow-up (concentration at 8-year – concentration at 16-year) and between 8-year and 24-year follow-up (concentration at 8-year – concentration at 24-year), with positive values indicating improvement of air quality.

Supplementary Table E5 Spearman correlations between reduction of air pollutants between age 8 to age 16 and age 8 to age 24

	$PM_{2.5}$ 8 to 16	PM_{10} 8 to 16	BC 8 to 16	NO_x 8 to 16	$PM_{2.5}$ 8 to 24	PM_{10} 8 to 24	BC 8 to 24	NO_x 8 to 24
$PM_{2.5}$ 8 to 16	1	0.92	0.83	0.76	0.71	0.60	0.73	0.49
PM_{10} 8 to 16		1	0.90	0.89	0.63	0.63	0.74	0.55
BC 8 to 16			1	0.98	0.67	0.66	0.90	0.64
NO_x 8 to 16				1	0.62	0.64	0.84	0.64
$PM_{2.5}$ 8 to 24					1	0.89	0.78	0.73
PM_{10} 8 to 24						1	0.81	0.92
BC 8 to 24							1	0.81
NO_x 8 to 24								1

$PM_{2.5}$, particulate matter with diameter ≤ 2.5 µm; PM_{10}, particulate matter with diameter ≤ 10 µm; BC, black carbon; NOx, nitrogen oxides.

Supplementary Table E6 Sensitivity analyses of the association between air quality improvement and differences in lung function growth from age eight to age 24 years

Sensitivity Analysis*	Air pollutants	Differences of FEV1 growth, ml/year (95%CI)	Differences of FVC growth, ml/year (95%CI)
Using the relative reduction of air pollution as exposure	$PM_{2.5}$	4.48 (1.54, 1.72)	9.25 (4.68, 13.83)
	PM_{10}	0.48 (-1.38, 2.33)	2.77 (-0.20, 5.73)
	BC	3.56 (0.57, 6.55)	7.69 (2.97, 12.40)
	NO_x	2.20 (0.37, 4.03)	4.81 (0.68, 8.94)
Additional adjusted for short-term air pollution exposure	$PM_{2.5}$	4.20 (0.97, 7.43)	9.50 (4.50, 14.49)
	PM_{10}	0.43 (-1.28, 2.13)	2.39 (-0.30, 5.08)
	BC	2.34 (0.08, 4.60)	5.21 (1.75, 8.68)
	NO_x	1.45 (-0.52, 3.43)	2.78 (-0.31, 5.89)
Using time-weighted average exposure between preceding and current follow-ups	$PM_{2.5}$	3.33 (0.70, 5.95)	10.39 (4.08, 16.69)
	PM_{10}	0.71 (-0.51, 1.93)	1.39 (-0.55, 3.34)
	BC	3.93 (1.34, 6.52)	7.33 (3.24, 11.42)
	NO_x	2.52 (0.10, 4.94)	4.55 (0.72, 8.38)
Using 8-year, 16-year, COVID-19* follow-up spirometry data	$PM_{2.5}$	2.65 (0.83, 4.48)	3.49 (0.48, 6.51)
	PM_{10}	0.59 (-0.29, 1.48)	1.23 (-0.33, 2.79)
	BC	2.18 (0.64, 3.72)	3.47 (1.00, 5.94)
	NO_x	1.85 (0.39, 3.31)	3.32 (1.05, 5.58)

Results were adjusted for age, sex, height, body mass index at age 8, municipality at birth, parental education level, parental occupation at birth, maternal smoking during pregnancy, environmental tobacco smoking and air pollution exposure at the first year of life, BMI at 16-, 24-year, active smoking at 16-, 24-year and education level at 24-year

*: Annual air pollution data at the residential addresses for 2020 were used for the COVID-19 follow-up (mean level: 3.79±0.4 μg/m^3 for PM2.5, 7.58±1.67 μg/m^3 for PM10, 0.21±0.05 μg/m^3 for BC, 9.0±5.3 μg/m^3 for NOx) to calculate the improved air quality.

Supplementary Table E7 Distribution of the reduced air pollution exposure during the study period stratified based on moving after 8-year follow-up

	16-year follow-up		24-year follow-up	
	Mover	Non-mover	Mover	Non-mover
Reduction of $PM_{2.5}$ from 8-year*, μg/m^3	1.65 (0.60)	1.69 (0.46)	3.00 (0.95)	3.00 (0.70)
Reduction of PM_{10} from 8-year*, μg/m^3	0.32 (1.39)	0.30 (1.23)	1.76 (2.02)	1.82 (1.18)
Reduction of BC from 8-year*, μg/m^3	0.13 (0.27)	0.16 (0.24)	0.38 (0.38)	0.38 (0.38)
Reduction of NO_x from 8-year*, μg/m^3	5.36 (6.87)	5.89 (5.83)	5.41 (10.20)	6.12 (6.36)

Data were presented as median (interquartile range). $PM_{2.5}$, particulate matter with diameter ≤ 2.5 μm; PM_{10}, particulate matter with diameter ≤ 10 μm; BC, black carbon; NOx, nitrogen oxides.
*: air pollution changes between the 8-year and 16-year follow-up (concentration at 8-year – concentration at 16-year) and between 8-year and 24-year follow-up (concentration at 8-year – concentration at 24-year), with positive values indicating improvement of air quality.

Supplemental Table E8 Association between improvement of air quality and differences in lung function growth stratified by study period

			Raw values as outcome		GLI z-score as outcome	
Study period	Air pollutants	Unit of change in exposure*, μg/m³	Difference in FEV_1 growth, ml/year (95%CI)	Difference in FVC growth, ml/year (95%CI)	Difference in FEV_1 growth, SD/year (95%CI)	Difference in FVC growth, SD/year (95%CI)
Year 8 to Year 16 (N=1224)	$PM_{2.5}$	1.59	103.70 (72.83, 134.57)	154.33 (102.81, 205.86)	0.07 (0.04, 0.10)	0.08 (0.06, 0.11)
	PM_{10}	0.16	2.21 (-1.57, 5.99)	4.11 (-2.23, 10.44)	0.01 (0.01, 0.02)	0.01 (0.01, 0.02)
	BC	0.11	29.46 (17.41, 41.52)	42.31 (22.11, 62.49)	0.03 (0.01, 0.05)	0.03 (0.01,0.05)
	NO_x	4.67	41.97 (25.32, 58.63)	64.92 (37.07, 92.78)	0.03 (0.00, 0.07)	0.05 (0.01,0.08)
Year 16 to Year 24 (N=837)	$PM_{2.5}$	1.27	2.49 (-0.06, 5.03)	6.46 (2.23, 10.69)	0.01 (-0.00, 0.03)	0.01 (0.00, 0.03)
	PM_{10}	2.01	1.06 (-2.39, 4.52)	3.54 (-1.50, 8.59)	-0.01 (-0.03, 0.00)	-0.02 (-0.03, 0.00)
	BC	0.38	2.31 (-0.94, 5.56)	5.64 (0.62, 10.65)	-0.00 (-0.02, 0.01)	0.001 (0.000, 0.003)
	NO_x	7.31	1.61 (-0.69, 3.91)	3.61 (0.40, 6.83)	-0.00 (-0.01, 0.01)	0.005 (0.000, 0.010)

Results were adjusted for age, sex, height, body mass index at age 8, municipality at birth, parental education level, parental occupation at birth, maternal smoking during pregnancy, environmental tobacco smoking and air pollution exposure at the first year of life, BMI at 16-, 24-year, active smoking at 16-, 24-year and education level at 24-year

*: air pollution changes between the 8-year and 16-year follow-up (concentration at 8-year – concentration at 16-year) and between 8-year and 24-year follow-up (concentration at 8-year – concentration at 24-year), with positive values indicating improvement of air quality.

Supplemental Table E9 Odds ratios for the associations between improved air quality and lower limit of normal (LLN) z-scores of FEV1 and FVC at 16-years and 24-years of age

Air pollutants	Odds ratios (95%CIs)	
	< LLN FEV_1	<LLN FVC
$PM_{2.5}$	0.90 (0.84, 0.97)	0.78 (0.70, 0.86)
PM_{10}	1.01 (0.97, 1.04)	1.00 (0.96, 1.05)
BC	0.96 (0.91, 1.00)	0.94 (0.89, 1.01)
NO_x	0.99 (0.96, 1.02)	1.01 (0.96, 1.07)

LLN: lower limit of normal; FEV_1: forced expiratory volume in 1s; FVC, forced vital capacity; $PM_{2.5}$, particulate matter with diameter ≤ 2.5 µm; PM_{10}, particulate matter with diameter ≤ 10 µm; BC, black carbon; NOx, nitrogen oxides. GLI, global lung initiative.

Odds ratios were adjusted for age, sex, height, body mass index at age 8, municipality at birth, parental education level, parental occupation at birth, maternal smoking during pregnancy, environmental tobacco smoking, air pollution exposure during the first year of life, BMI at 16-, 24-year, active smoking at 16-, 24-year and education level at 24-year. Odds ratios were presented as per interquartile range change for air pollution reduction: 2.19 µg/m^3 for $PM_{2.5}$, 1 µg/m^3 for PM_{10}, 0.28 µg/m^3 for BC, 6.17 µg/m^3 for NO_x.

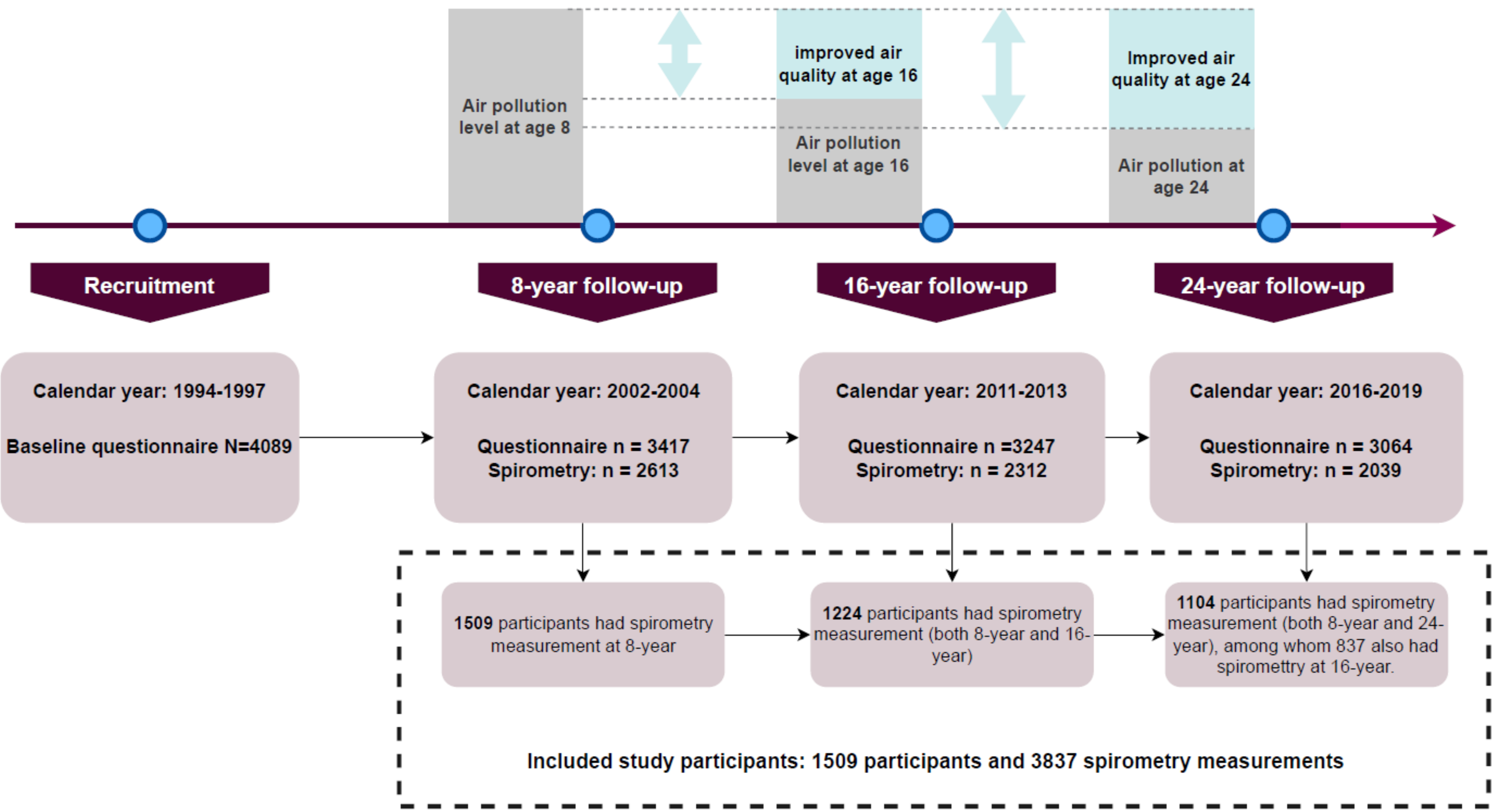

Supplementary Figure E1. Flow chart of the study timeline. Information in the dashed square presented the number of participants and spirometry measurements included in the analysis.

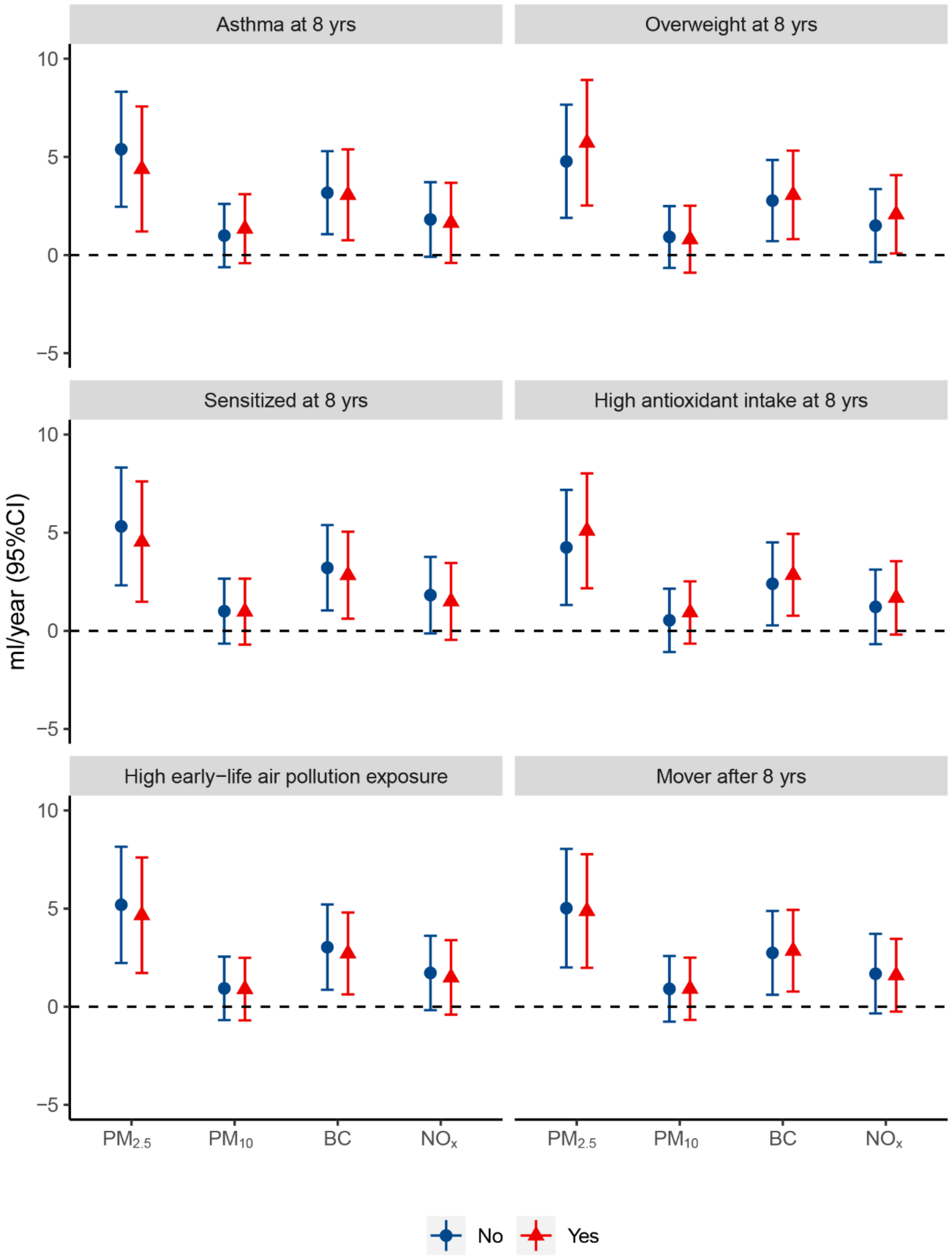

Supplementary Figure E2 Subgroup analyses of the association between improvement of air quality and differences in FEV_1 growth from age eight to age 24.

Results were adjusted for age, sex, height, body mass index at age 8, municipality at birth, parental education level at birth, parental occupation at birth, maternal smoking during pregnancy, environmental tobacco smoking, air pollution exposure during the first year of life, , BMI at 16-, 24-year, active smoking at 16-, 24-year and education level at 24-year.

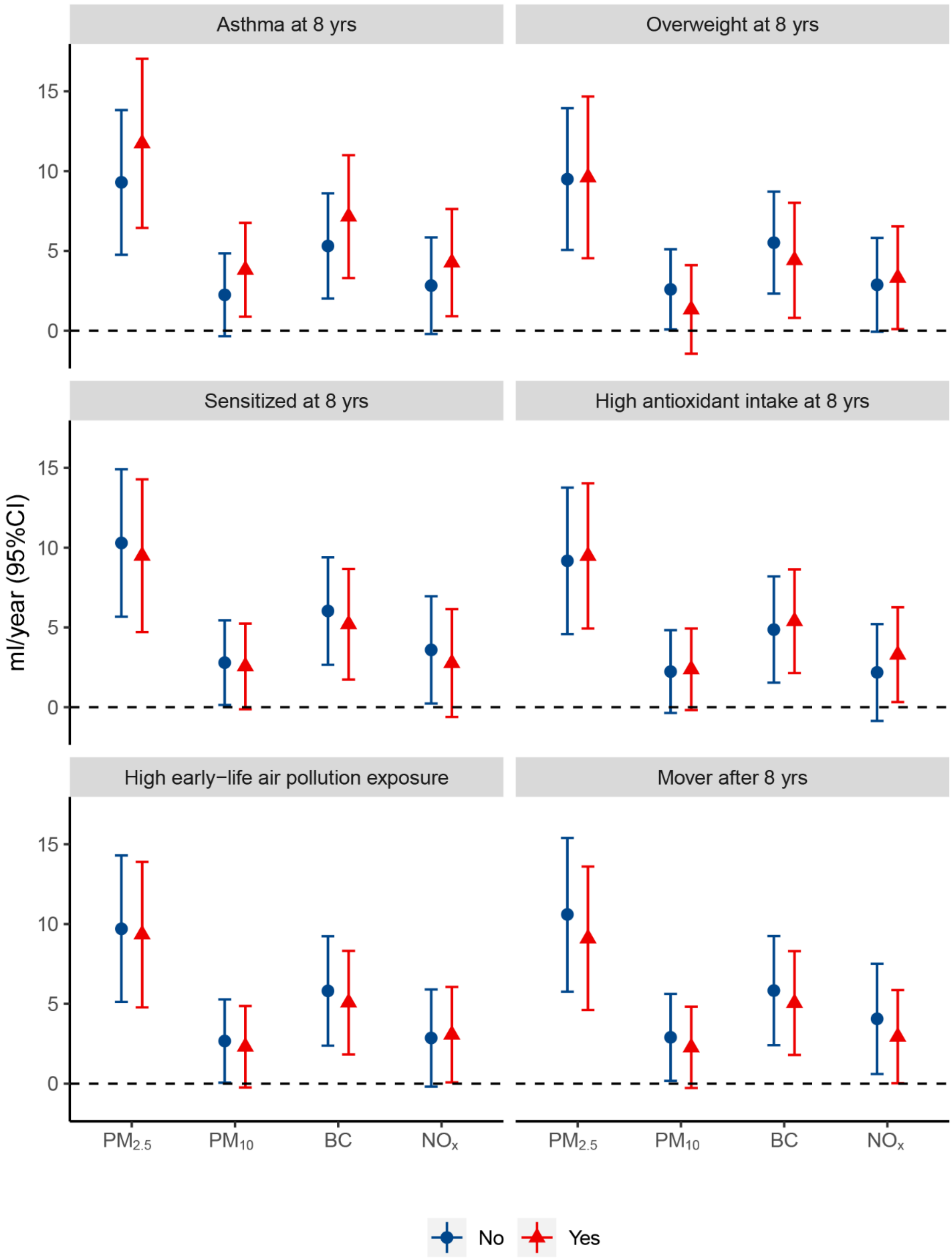

Supplementary Figure E3 Subgroup analyses of the association between improvement of air quality and differences in FVC growth from age eight to age 24.

Results were adjusted for age, sex, height, body mass index at age 8, municipality at birth, parental education level at birth, parental occupation at birth, maternal smoking during pregnancy, environmental tobacco smoking, air pollution exposure during the first year of life, BMI at 16-, 24-year, active smoking at 16-, 24-year and education level at 24-year.

References

1. Ekstrom S, Andersson N, Lovquist A, *et al*. COVID-19 among young adults in Sweden: self-reported long-term symptoms and associated factors. *Scand J Public Health* 2021: 14034948211025425.
2. Bjorkander S, Du L, Zuo F, *et al*. SARS-CoV-2-specific B- and T-cell immunity in a population-based study of young Swedish adults. *The Journal of allergy and clinical immunology* 2021.
3. Miller MR, Hankinson J, Brusasco V, *et al*. Standardisation of spirometry. *European respiratory journal* 2005: 26(2): 319-338.
4. Mogensen I, Hallberg J, Björkander S, *et al*. Lung function before and after COVID-19 in young adults: A population-based study. *Journal of Allergy and Clinical Immunology: Global* 2022.
5. Gruzieva O, Merid SK, Gref A, *et al*. Exposure to Traffic-Related Air Pollution and Serum Inflammatory Cytokines in Children. *Environ Health Perspect* 2017: 125(6): 067007.
6. Omstedt G, Bringfelt B, Johansson C. A model for vehicle-induced non-tailpipe emissions of particles along Swedish roads. *Atmospheric environment* 2005: 39(33): 6088-6097.
7. Denby BR, Sundvor I, Johansson C, *et al*. A coupled road dust and surface moisture model to predict non-exhaust road traffic induced particle emissions (NORTRIP). Part 1: Road dust loading and suspension modelling. *Atmospheric Environment* 2013: 77: 283-300.
8. Denby BR, Sundvor I, Johansson C, *et al*. A coupled road dust and surface moisture model to predict non-exhaust road traffic induced particle emissions (NORTRIP). Part 2: Surface moisture and salt impact modelling. *Atmospheric Environment* 2013: 81: 485-503.
9. Pinart M, Benet M, Annesi-Maesano I, *et al.* Comorbidity of eczema, rhinitis, and asthma in IgE-sensitised and non-IgE-sensitised children in MeDALL: a population-based cohort study. *Lancet Respir Med* 2014: 2(2): 131-140.
10. Wickman M, Asarnoj A, Tillander H, *et al*. Childhood-to-adolescence evolution of IgE antibodies to pollens and plant foods in the BAMSE cohort. *The Journal of allergy and clinical immunology* 2014: 133(2): 580-582.
11. Sdona E, Hallberg J, Andersson N, *et al*. Dietary antioxidant intake in school age and lung function development up to adolescence. *Eur Respir J* 2020: 55(2).
