# Supplementary material for: Associations of improved air quality with lung function growth from childhood to adulthood: the BAMSE study
Source: Eur Respir J. 2023 May 4;61(5):2201783. doi: 10.1183/13993003.01783-2022 (PMC10160798; doi:10.1183/13993003.01783-2022)
Supplement: Supplementary file 2 [file ERJ-01783-2022.Shareable.pdf]

EUROPEAN RESPIRATORY JOURNAL
ORIGINAL RESEARCH ARTICLE
Z. YU ET AL.

SHAREABLE PDF

Associations of improved air quality with lung function growth from childhood to adulthood: the BAMSE study

Zhebin Yu[1], Simon Kebede Merid[2], Tom Bellander[1,3], Anna Bergström[1,3], Kristina Eneroth[4], Antonios Georgelis[1,3], Jenny Hallberg[2,5], Inger Kull[2,5], Petter Ljungman[1,6], Susanna Klevebro[2,5], Massimo Stafoggia[1,7], Gang Wang[1,2], Göran Pershagen[1,3], Olena Gruzieva[1,3] and Erik Melén[2,5]

[1]Institute of Environmental Medicine, Karolinska Institutet, Stockholm, Sweden. [2]Department of Clinical Sciences and Education, Södersjukhuset, Karolinska Institutet, Stockholm, Sweden. [3]Centre for Occupational and Environmental Medicine, Region Stockholm, Stockholm, Sweden. [4]SLB-analys, Environment and Health Administration, Stockholm, Sweden. [5]Sachs' Children and Youth Hospital, Södersjukhuset, Stockholm, Sweden. [6]Department of Cardiology, Danderyd Hospital, Stockholm, Sweden. [7]Department of Epidemiology, Lazio Regional Health Service, Rome, Italy.

Corresponding author: Erik Melén (erik.melen@ki.se)

Check for updates

Shareable abstract (@ERSpublications)
Decrease in ambient air pollution exposure, even at relatively low levels, is associated with improvements in lung function growth from childhood to early adulthood https://bit.ly/3jAFZTq

Cite this article as: Yu Z, Merid SK, Bellander T, *et al.* Associations of improved air quality with lung function growth from childhood to adulthood: the BAMSE study. *Eur Respir J* 2023; 61: 2201783 [DOI: 10.1183/13993003.01783-2022].

This single-page version can be shared freely online.

Copyright ©The authors 2023.

This version is distributed under the terms of the Creative Commons Attribution Licence 4.0.

This article has an editorial commentary: https://doi.org/10.1183/13993003.00345-2023

Received: 13 Sept 2022
Accepted: 29 Jan 2023

Abstract

Background The beneficial effect of improving air quality on lung function development remains understudied. We assessed associations of changes in ambient air pollution levels with lung function growth from childhood until young adulthood in a Swedish cohort study.

Methods In the prospective birth cohort BAMSE (Children, Allergy, Environment, Stockholm, Epidemiology (in Swedish)), spirometry was conducted at the 8-year (2002–2004), 16-year (2011–2013) and 24-year (2016–2019) follow-ups. Participants with spirometry data at 8 years and at least one other measurement in subsequent follow-ups were included (1509 participants with 3837 spirometry measurements). Ambient air pollution levels (particulate matter with diameter $\leqslant 2.5\ \mu m$ ($PM_{2.5}$), particulate matter with diameter $\leqslant 10\ \mu m$ (PM_{10}), black carbon (BC) and nitrogen oxides (NO_x)) at residential addresses were estimated using dispersion modelling. Linear mixed effect models were used to estimate associations between air pollution exposure change and lung function development.

Results Overall, air pollution levels decreased progressively during the study period. For example, the median (interquartile range (IQR)) level of $PM_{2.5}$ decreased from 8.24 (0.92) $\mu g \cdot m^{-3}$ during 2002–2004 to 5.21 (0.67) $\mu g \cdot m^{-3}$ during 2016–2019. At the individual level, for each IQR reduction of $PM_{2.5}$ the lung function growth rate increased by 4.63 (95% CI 1.64–7.61) mL per year ($p<0.001$) for forced expiratory volume in 1 s and 9.38 (95% CI 4.76–14.00) mL per year ($p<0.001$) for forced vital capacity. Similar associations were also observed for reductions of BC and NO_x. Associations persisted after adjustment for potential confounders and were not modified by asthma, allergic sensitisation, overweight, early-life air pollution exposure or dietary antioxidant intake.

Conclusions Long-term reduction of air pollution is associated with positive lung function development from childhood to young adulthood.
